# A novel *DNM2* variant associated with centronuclear myopathy: a case report

**DOI:** 10.3389/fgene.2025.1559773

**Published:** 2025-04-07

**Authors:** Martina Rimoldi, Daniele Velardo, Simona Zanotti, Michela Ripolone, Roberto Del Bo, Patrizia Ciscato, Laura Napoli, Stefania Corti, Giacomo Pietro Comi, Dario Ronchi

**Affiliations:** ^1^ Fondazione IRCCS Ca’ Granda Ospedale Maggiore Policlinico, Neuromuscular and Rare Disease Unit, Milan, Italy; ^2^ Dino Ferrari Center, Department of Pathophysiology and Transplantation, University of Milan, Milan, Italy; ^3^ Fondazione IRCCS Ca’ Granda Ospedale Maggiore Policlinico, Neurology Unit, Milan, Italy

**Keywords:** *DNM2*, centronuclear myopathy, myopathy, dynamin-2, neuromuscular disease

## Abstract

*DNM2* encodes the dynamin-2 protein, a GTPase involved in clathrin-mediated endocytosis and other membrane trafficking pathways. The dynamin-2 protein is composed of several functional domains, including a GTPase domain, a middle domain, a pleckstrin homology (PH) domain, a GTPase effector domain (GED), and a proline-rich domain. Monoallelic variants in *DNM2* are associated with Charcot–Marie–Tooth disease and a rare form of congenital centronuclear myopathy (CNM). Several *DNM2* variants have been reported in patients with CNM, typically presenting with mild and slowly progressive symptoms. We report the case of a 47-year-old man with *DNM2*-related myopathy, who presented with progressive muscle weakness starting at the age of 40 years. Clinical exome sequencing revealed the presence of a heterozygous *DNM2* variant c.1726G>A, p.(Glu576Lys). This variant, previously unreported, is located in the PH domain of the protein. Muscle biopsy findings showed several fibers with central nuclei, sometimes multiple. In addition, occasional centronucleated fibers showed a radial distribution of sarcoplasmic strands. This study expands the clinical and genetic repertoire of DNM2-related myopathy.

## 1 Introduction

The *DNM2* gene encodes a protein called dynamin-2, a component of the dynamin family of GTPases, which plays a crucial role in various cellular processes, particularly in endocytosis and membrane trafficking (Antonny et al., 2016; Ferguson and De Camilli, 2012).

The *DNM2* gene is located on the short arm of chromosome 19 (19p13.2). It spans approximately 59 kilobases and consists of 22 exons. The dynamin-2 protein includes a GTPase domain, a middle domain, a pleckstrin homology (PH), a GTPase effector domain (GED), and a proline-rich domain (Hayes et al., 2022).

Dynamin-2 plays a crucial role in clathrin-mediated endocytosis, facilitating the fission of clathrin-coated vesicles from the plasma membrane. Dynamin-2 is also involved in other membrane trafficking pathways, including receptor recycling, synaptic vesicle recycling, and the maintenance of organelle morphology (Puri et al., 2020).

Monoallelic variants in the *DNM2* are associated with heterogeneous clinical presentations, including dominant intermediate Charcot–Marie–Tooth disease (CMTDIB, MIM 606482), dominant axonal Charcot–Marie–Tooth disease (CMT2M, MIM 606482), and a form of centronuclear myopathy (CNM, MIM 160150), characterized by muscle weakness. In *DNM2*-related CNM patients, muscle biopsy usually shows disorganization of myofibers and centralized nuclei (Jungbluth et al., 2008; Romero, 2010; Biancalana et al., 2018). Ultrastructural studies reveal disorganized transverse tubules and triads (Bitoun et al., 2005; Al-Qusairi e Laporte, 2011).

Pathogenic variants in *DNM2* were associated with myopathic clinical presentations ranging from severe neonatal onset to adult-onset disorders with a milder phenotype (Böhm et al., 2012; Catteruccia et al., 2013; Casar-Borota et al., 2015; Hohendahl, Roux, and Galli, 2016; Biancalana et al., 2018; Vandersmissen et al., 2018; Westra et al., 2019; Reumers et al., 2021; Hayes et al., 2022). The prognosis of CNM can vary widely among affected individuals. Some patients may experience a relatively stable course with minimal progression of muscle weakness, while others may develop a more severe and rapidly progressive form of the disease. Regular follow-up and continuous monitoring of disease progression are essential to adapting patient management (Susman et al., 2010; Hayes et al., 2022).

The majority of the reported *DNM2* variants result in amino acid changes in the PH and stalk domains.

In this report, we present the case of a 47-year-old man affected with *DNM2*-related myopathy associated with a novel missense variant in the PH domain of dynamin-2.

## 2 Methods

The study was approved by the institutional review board of the Fondazione IRCCS Ca’ Granda Ospedale Maggiore Policlinico. The patients provided written informed consent for all aspects of the study. Genomic DNA was extracted from peripheral blood samples taken from both the patient and his mother. The QIAsymphony automated nucleic acid extraction platform (QIAGEN) was utilized for this purpose.

Clinical exome sequencing was performed on the affected proband, starting with 100 ng of high-quality DNA. Agilent SureSelectXT Clinical Focused Exome library preparation and target enrichment kits were used. The libraries underwent paired-end sequencing on a NextSeq 2000 Illumina platform. The variants included in the generated VCF files were annotated based on the genome assembly of hg19 and classified using an internal analysis pipeline and the bioinformatic tool eVai Expert Variant Interpreter v2.7.

PCR amplification, followed by Sanger sequencing (using Thermo Fisher Scientific BigDye Terminator v3.1) on an ABI Prism 3130 automated DNA analyzer, was performed to validate the candidate variant in the *DNM2* gene on both the proband and his mother.

### 2.1 Histological and histochemical analysis

Tissue specimens were frozen in isopentane-cooled liquid nitrogen and processed according to the standard techniques. For histological analysis, 8-µm-thick cryosections were selected and processed for routine staining with hematoxylin and eosin (H&E), modified Gomori trichrome (MGT), myosin ATPase (pH 9.4-4.6-4.3), cytochrome c oxidase (COX), succinate dehydrogenase (SDH), phosphatase acid, NADH dehydrogenase, Oil Red O, and periodic acid–schiff (PAS).

### 2.2 Immunofluorescence

For immunofluorescence staining, 8-μm-thick muscle cryosections were fixed with acetone for 3 min, washed three times with PBS, and blocked with 1% BSA diluted in PBS for 30 min at RT. Muscle sections were incubated overnight at 4°C with one of the following antibodies: C5b-9 (1:50, mouse monoclonal, Dako, Glostrup, Denmark), αB-crystallin (1:100, rabbit polyclonal, Sigma), NH-Dystrophin (1:10 mouse monoclonal, Novocastra, Newcastle upon Tyne, United Kingdom), myotilin (1:10, mouse monoclonal, Novocastra), spectrin (1:100, rabbit polyclonal, Sigma), and p62 (1:100, rabbit polyclonal, Abcam, Cambridge, United Kingdom), lamp-2 (1:200, rabbit polyclonal, Sigma). Caveolin-3 (1:1,000, mouse monoclonal, BD Biosciences, Franklin Lakes, NJ, United States) and laminin-α2 (1:200, rabbit polyclonal, Thermo Fisher Scientific, Waltham, MA, United States) were used for membrane staining. Following three washes with PBS, sections were incubated with the secondary antibody goat anti-mouse Alexa-488 or goat anti-rabbit Alexa-596 (1:1,000; Thermo Fisher Scientific) for 2 hours at RT. Finally, after three washes with PBS, slides were mounted with the anti-fading reagent Fluoromount (Thermo Fisher Scientific).

### 2.3 Electron microscopy

For ultrastructural examination, a small part of the muscle sample was fixed in 2.5% glutaraldehyde at pH 7.4 (Electron Microscopy Sciences EMS, Hatfield, PA, United States) for 1 h at room temperature and overnight at 4°C; the specimens were washed in 0.1 M, pH 7.4 cacodylate buffer (EMS), and post-fixed for 1 h in 2% osmium tetroxide (EMS). Next, the specimens were dehydrated using a graded series of ethyl alcohol and embedded in Epon resin (EMS). Finally, ultrathin sections (80-nm-thick slices) were prepared using an Ultramicrotome PowerTome XL (RMC, Tucson, AZ, USA). The grids, stained with 0.5% lead citrate (EMS) and uranyl acetate replacement/methanol 1:1 (EMS), were examined using a Hitachi HT7800 Transmission Electron Microscope (Hitachi, Japan).

## 3 Case description and results

**FIGURE 1:**
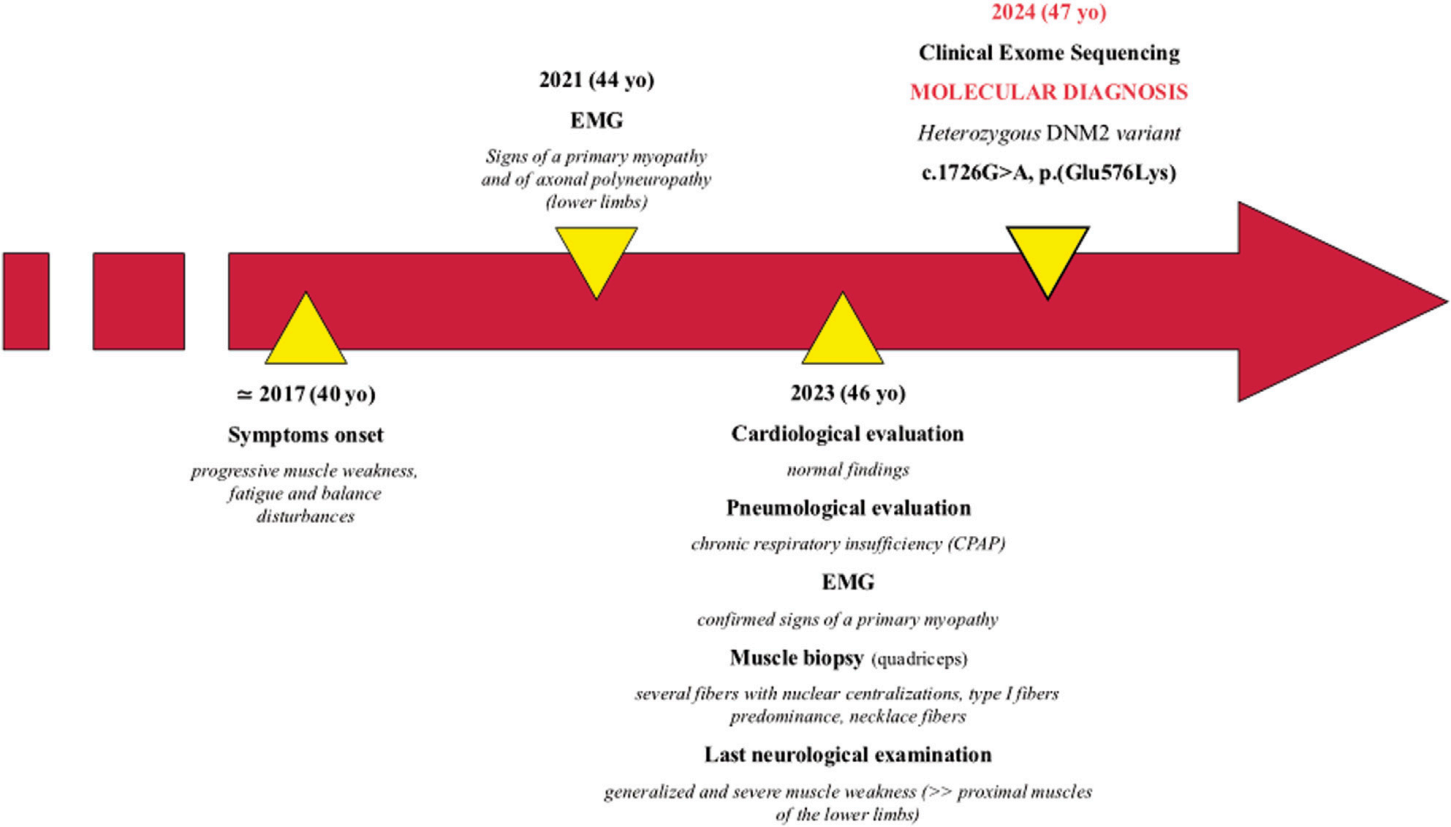
Patient’s timeline.

The patient is a 47-year-old man, first born from non-consanguineous healthy parents of Brazilian origin (Figure 1). Family history is negative for neuromuscular disorders.

He presented with progressive muscle weakness, fatigue, and balance disturbances since his 40s, which were reported as difficulty in walking and climbing stairs. The symptoms mainly affected his lower limbs. During his last hospitalization at 46 years of age, the proband showed generalized and severe muscle weakness, with predominant involvement of the proximal muscles of the lower limbs. Needle electromyographic examination showed a myopathic pattern (motor unit action potentials of reduced duration and amplitude and early recruitment pattern) pointing toward a pathological process with a primary origin from the muscle. A nerve conduction study showed the presence of mild sensory–motor axonal polyneuropathy in the lower limbs in the absence of specific clinical symptoms.

Currently, unassisted ambulation is possible, even though mild waddling gait is present. The patient is not able to stand up from a squatting position without external aids.

Cardiological evaluation performed at 46 years, including Holter electrocardiography and echocardiography, was normal.

A thoracic computed tomography scan showed subpleural interstitial thickening in the lower lobes bilaterally, without pleural effusions. The patient was thereby diagnosed with obstructive sleep apnea syndrome with chronic respiratory insufficiency, conditioning the introduction of continuous positive airway pressure (CPAP) therapy. Creatine kinase dosage tests returned normal results.

**FIGURE 2:**
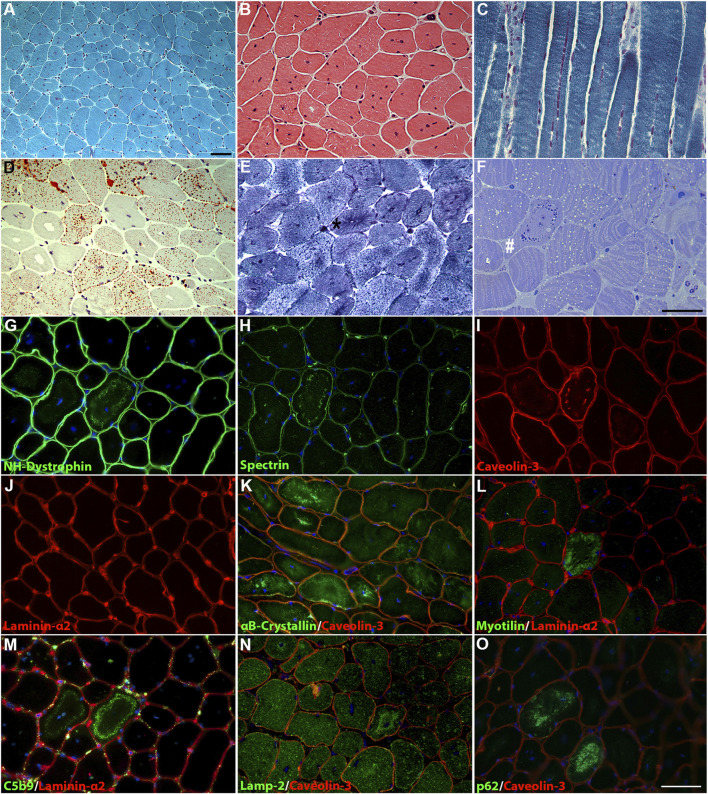
Histological findings in the patient’s muscle. **(A)** MGT and **(B)** H&E staining revealed fiber size variability and centronuclear fibers. **(C)** Longitudinal MGT section showed numerous nuclei accumulating in rows. **(D)** Oil red O staining revealed increased content of lipid droplets in numerous fibers. **(E)** NADH dehydrogenase staining showed a radial strand fiber (asterisk). **(F)** Semi-thin sections showed the presence of a “necklace fiber” (hashtag) and numerous fibers with an increase in lipid contents. Scale bar, 20 µm. Immunofluorescence staining showed positivity for the membrane proteins NH-dystrophin **(G)**, spectrin **(H)**, and caveolin-3 **(I)** associated with the cytoplasmic vacuolar structures, except for laminin-α2 **(J)**. Altered distribution for αB-crystallin **(K)** in several fibers and increased deposition of myotilin **(L)** in necklace fibers. The cytoplasmic vacuolar structures showed marked positivity for C5b9 **(M)**, lamp-2 **(N)**, and p62 **(O)**. DAPI for counterstaining of nuclei. Scale bar, 20 µm.

At 46 years, a quadriceps muscle biopsy was performed. Histological analysis revealed a primary muscular involvement and characteristic features associated with *DNM2*-related myopathy. MGT and H&E showed mild variability in fiber size and numerous fibers containing multiple centralized nuclei (Figures 2A, B), accumulating in rows when observed in longitudinal sections (Figure 2C). Occasionally, fibers showed a radial distribution of sarcoplasmic strands. Oil red O staining demonstrated an increase in the lipid content in some type I fibers (Figure 2D). NADH staining showed the presence of radial strand fiber, and semi-thin sections revealed the presence of necklace cytoplasmic bodies (Figures 2E, F). Type I fibers showed mild hypotrophy and were slightly more prevalent than type II fibers (58.3% versus 41.6%, respectively), with a moderate shift in the distribution pattern compared to that of the control quadriceps muscle (36% versus 64%, respectively).

Immunofluorescence showed positivity for the membrane proteins NH-dystrophin, spectrin, and caveolin-3, indicating a membrane lining of these vacuoles, but positive staining for laminin-α2 was not observed (Figures 2G–J). Several fibers showed altered staining for αB-crystalline, with marked protein accumulation in numerous fibers (Figure 2K). The “necklace fibers” showed strong positivity for myotilin (Figure 2L). Strong positivity associated with these vacuolar structures was detected for C5b-9 (Figure 2M); in addition, lamp-2 (Figure 2N) and p62 (Figure 2O) positivity were detected in the same vacuoles, suggesting a lysosomal origin. Similar findings were previously reported by Rinnenthal et al. (2018) in two patients from two independent families carrying the *DNM2* variant c.1106G>A, p.(Arg369Gln).

**FIGURE 3:**
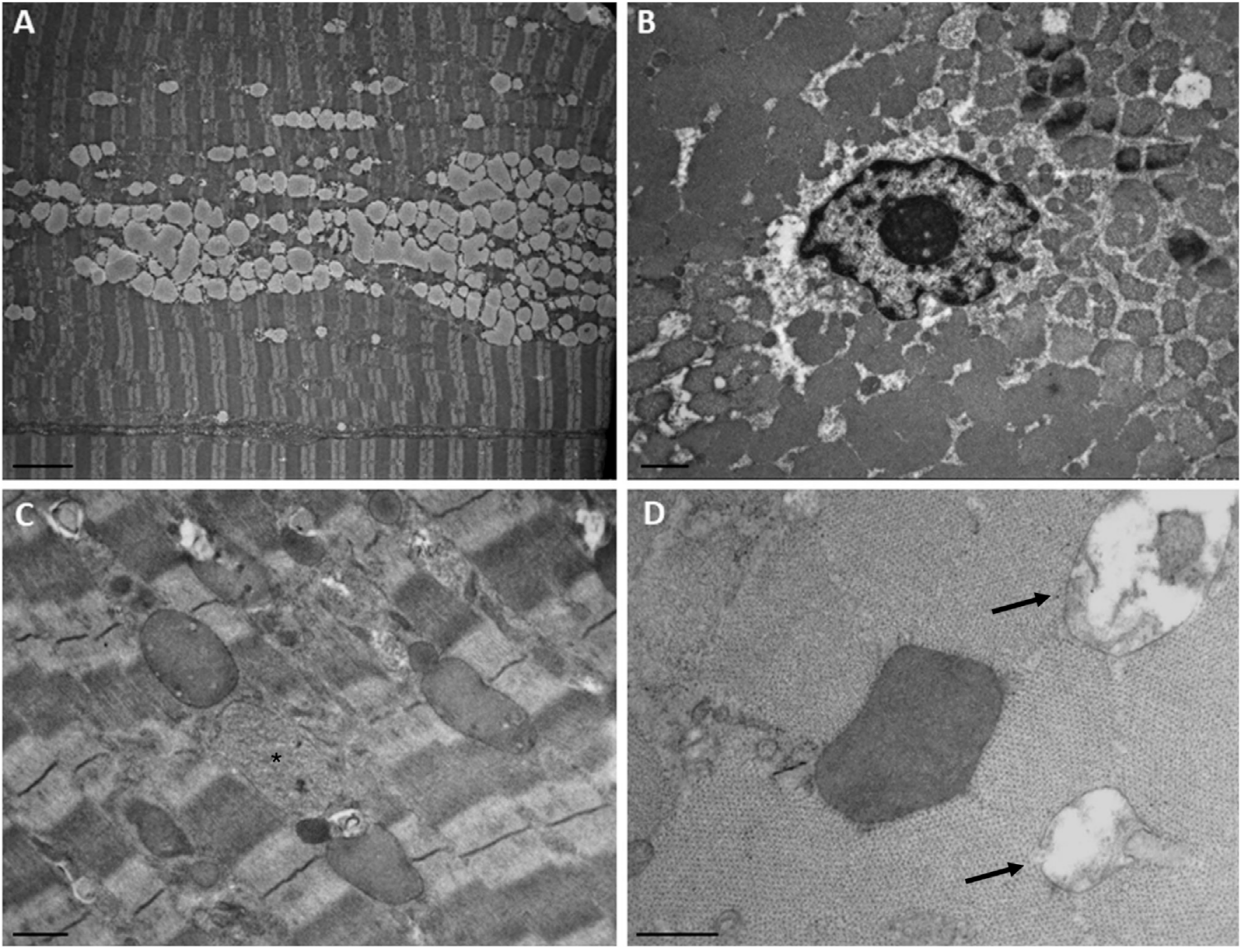
Ultrastructural findings in the patient’s muscle. **(A)** Numerous confluent lipid droplets in the center of the fiber. **(B)** Transverse section showed the nucleus in the center of the fiber. **(C)** Inclusions containing strongly electron-dense or finely granular material (asterisk). **(D)** Higher magnification of an electron-dense body and degraded mitochondria (arrows). Scale bar, A, 5 µm; B and C, 1 μm; D, 0.5 µm.

Ultrastructural analysis (Figure 3) revealed numerous central nuclei, a moderate increase in lipid droplets, and lipofuscin granules. Occasionally, altered mitochondria were observed, showing cristae rarefaction and matrix loss. In some fibers, round vacuoles surrounded by a membrane and containing a strongly electron-dense, homogeneous granular material were observed. A second type of inclusion containing less electron-dense material was rarely observed. These regions likely correspond to those observed in semi-thin sections, where some fibers exhibit vacuoles that were localized in a circular pattern, approximately two-thirds of the distance from the center of the fiber to the sarcolemma, appearing as necklace-like fibers. We did not observe any alterations in the T-tubules and triads.

**FIGURE 4:**
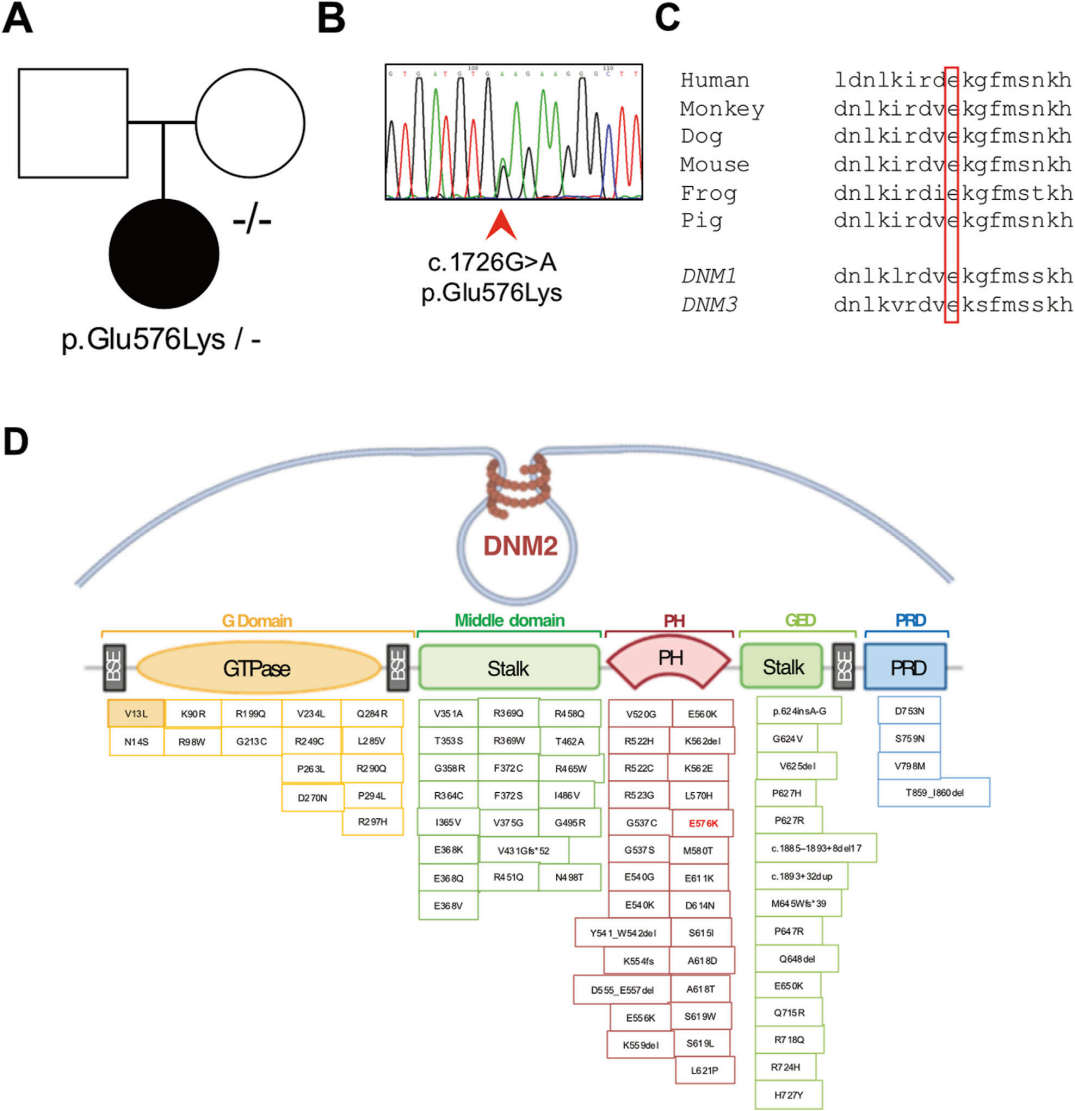
Molecular studies. **(A)** Pedigree of the family (affected member is represented by a black symbol) and **(B)** electropherogram showing the presence of the novel heterozygous c.1726G>A p.(Glu576Lys) variant detected in the proband’s genomic DNA. **(C)** Conservation of the affected amino acidic positions across species and among human homolog genes *DNM1* and *DNM3*. **(D)** Schematic representation of the *DNM2* gene containing the identified pathogenic variations. The p.(Glu576Lys) change is indicated in red (E576K).

Clinical exome sequencing revealed the presence of the heterozygous chr19:10930710-G-A substitution (GrCh37) corresponding to the c.1726G>A, p.(Glu576Lys) variant in the *DNM2* gene (NM_001005360.2). The variant was confirmed by Sanger sequencing in the proband, whereas it was absent in the patient’s healthy mother (Figures 4A, B). A DNA sample from the patient’s father was not available, preventing the conclusive determination of a *de novo* origin for the variant. This variant is absent in the reference databases, predicted to be pathogenic by computational prediction tools (Supplementary Table S2), and classified as a variant of uncertain significance (VUS) according to the ACMG criteria (Richards et al., 2015). The variant affects a codon that is conserved across species and in the human *DNM1* and *DNM3* homolog genes (Figure 4C). The p.(Glu576Lys) variant is located in the PH domain of the protein, like most of the other *DNM2* variants that are reported as pathogenic (Figure 4D).

## 4 Discussion and conclusion

Despite the wide clinical variability, patients affected with *DNM2*-related congenital CNM often exhibit late-childhood, early adolescence, or adulthood-onset forms, although there are instances of *DNM2*-related CNM presenting as severe congenital myopathy. Common symptoms include slowly progressive skeletal muscle weakness, mainly involving the distal and axial muscles (Fischer et al., 2006). However, the involvement of the facial and bulbar muscles might impair the capacity of swallowing and speaking (bulbar weakness), while restrictive ventilatory impairment, with mild to severe forced vital capacity reduction, may require nocturnal non-invasive ventilation. Therefore, *DNM2*-related pathology is insidious and potentially life-threatening, even in patients presenting a late-onset, slowly progressing disease course (Bitoun et al., 2009; Catteruccia et al., 2013; Biancalana et al., 2018; Hayes et al., 2022; Ogasawara and Nishino, 2023). In addition to these “myopathic” findings, recent studies have also reported a mixed phenotype, with a coexistence of neuropathic signs, in terms of axonal peripheral nerve involvement evidenced at nerve conduction velocity (NCV) studies (Reumers et al., 2021). These findings highlight the partial clinical overlap between *DNM2*-related CNM and Charcot–Marie–Tooth neuropathy (Echaniz-Laguna et al., 2007). Regardless of the age at which symptoms appear, this condition progressively worsens, impacting independent ambulation: most of the patients perceive their condition as progressively deteriorating and require walking assistance during adulthood, if not earlier (Hayes et al., 2022).

Pathologically, *DNM2*-related centronuclear myopathy is usually characterized by centralized nuclei, predominance of type 1 muscle fibers, and radial distribution of sarcoplasmic strands (RSS) (Jeub et al., 2008; Romero, 2010; Park et al., 2014; Abath Neto et al., 2015). Nonetheless, in instances of severe cases, most of the fibers exhibit centrally located hyperintense responses surrounded by a faint peripheral halo, without typical RSS. This closely resembles the observations seen in individuals afflicted with XLMTM, an X-linked myotubular myopathy stemming from mutations in the *MTM1* (myotubularin 1) gene. In a single study, authors also reported evidence of necklace fibers at muscle biopsy, potentially suggesting a shared pathogenic pathway in centronuclear myopathies caused by both *DNM2* and *MTM1* gene mutations (Casar-Borota et al., 2015).

The patient’s muscle biopsy displayed several fibers with nuclear centralization, a characteristic feature in *DNM2*-mutated subjects, along with slight type I fiber predominance and the presence of necklace fibers (Romero, 2010).

The patient we are reporting presented with progressive skeletal muscle weakness, predominantly affecting the proximal muscles, sparing the facial and bulbar muscles. Disease onset was in his first adulthood, and the course is currently slowly progressive. He also required assisted ventilation from the age of 46 years old. He did not display ptosis, eye movement disorders, speech difficulties, or orofacial malformations, which have long been considered typical features of *DNM2*-related clinical presentation. However, recently published case series now acknowledge the existence of a wide phenotypic spectrum of *DNM2* myopathy. These additional manifestations are not consistently observed in all the patients in contrast to muscle weakness, fatigue, and exercise intolerance, which are described in almost every *DNM2*-mutated myopathic patient (Hayes et al., 2022; Reumers et al., 2021).

Since the first report (Bitoun et al., 2005), 76 different variants have been reported in *DNM2*, with half of them described as causative for CNM (Supplementary Table S1). In a single case, authors reported a patient affected with CNM carrying compound heterozygous *DNM2* mutations: despite his genotype, the subject displayed milder clinical manifestations than subjects carrying biallelic variants in *DNM2*, which are usually associated with the “lethal congenital contracture syndrome” (MIM #615368) (Chen et al., 2015). The majority of the reported pathogenic *DNM2* variants consist of missense changes situated within the PH and stalk domains. Recent studies (Böhm et al., 2012; Fujise et al., 2022; Hayes et al., 2022) have demonstrated that patients with mutations located at the autoinhibitory interface between the PH and middle domains, particularly p.(Glu368Lys), p.(Phe372Ser), p.(Ala618Asp), and p.(Ser619Leu), tend to exhibit more severe phenotypes characterized by earlier onset and faster progression of motor signs and respiratory difficulties. All these disease-causing substitutions are proposed to release autoinhibition of dynamin-2 and promote protein oligomerization, thus providing support for the “gain-of-function” mechanism advanced for *DNM2*-related CNM (Ogasawara and Nishino, 2023), based on their specific locations.

The variant detected in the patient we are reporting (p.Glu576Lys) affects a highly conserved position and is absent from population databases. However, the lack of functional experiments addressing the DNM2 transcript and protein stability or the evaluation of DNM2 GTPase activity prevents us from drawing a conclusion about the pathogenicity of this variant that should be considered of uncertain significance.

Currently, there is no specific treatment available for *DNM2*-related CNM. Management is primarily focused on symptom alleviation and improving quality of life. This may involve a multidisciplinary approach, including physiotherapy to maintain muscle strength and mobility, occupational therapy to assist with activities of daily living, and the use of assistive devices as needed. However, promising therapies are being developed for this debilitating disease (Hayes et al., 2022). Recent studies using a mouse model of *DNM2*-related centronuclear myopathy have shown that molecular strategies aiming to reduce mutant dynamin-2 levels through the systemic delivery of viral vectors or the intramuscular administration of antisense oligonucleotides can improve the phenotype (Buono et al., 2018; de Carvalho Neves et al., 2023; Trochet et al., 2022). Based on these promising findings, a clinical trial using DYN101, an antisense oligonucleotide targeting *DNM2* pre-mRNA, was started in 2020 but was recently stopped because of liver enzyme elevations and low platelet counts in treated patients.

Further research is needed to explore new avenues for advancing the understanding and therapeutic development for this rare genetic muscle disorder. The identification of pathogenic *DNM2* variants in clinically affected subjects through NGS-based sequencing is crucial for accelerating patient and potential inclusion in future clinical trials.

## Data Availability

The datasets presented in this article are not readily available because of ethical and privacy restrictions. Requests to access the datasets should be directed to the corresponding author.
