# Supplementary material for: A novel DNM2 variant associated with centronuclear myopathy: a case report
Source: Front Genet. 2025 Apr 7;16:1559773. doi: 10.3389/fgene.2025.1559773 (PMC12010121; doi:10.3389/fgene.2025.1559773)
Supplement: Supplementary file 1 [file DataSheet1.pdf]

A novel DNM2 variant associated with centronuclear myopathy: a case report

Supplementary Material

Supplementary Table 1. List of the variants so far reported in DNM2, their localization in the Dynamin-2 protein and the associated phenotypes. CNM: centronuclear myopathy; HNM: Hereditary motor neuropathy; dHMN: distal Hereditary motor neuropathy; CMT: Charcot-Marie-Tooth; CMTDIB-Charcot-Marie-Tooth disease, dominant intermediate B. LOVD (Leiden Open variation Database (https://databases.lovd.nl/shared/variants/DNM2/unique)

Reference	DNM2 variant	Domain	Phenotype
Bitoun et al. 2005	c.1105C>T, p.(Arg369Trp) (R369W)	middle	CNM
	c.1393C>T, p.(Arg465Trp) (R465W)	middle	CNM
Bitoun et al. 2005; Ganaraja Valakunja et al. 2024	c.1102G>A, p.(Glu368Lys) (E368K)	middle	CNM; CMTDIB
Bitoun et al. 2005; Antoniadi et al. 2015	c.1106G>A, p.(Arg369Gln) (R369Q)	middle	CNM (Bitoun); HNM (Antoniadi)
Zuchner at al. 2005	c.1672A>G, p.(Lys562Glu) (K562E)	PH	CMT
	c.1672_1674delAAG, p.(Lys562del) (K562del)	PH	CMT
	p.(Lys554fs) (K554fs)	PH	CMT
	p.(Asp555_Glu557del) (D555_E557del)	PH	CMT
Bitoun et al. 2007	c.1852G>A, p.(Ala618Thr) (A618T)	PH	CNM
	c.1856C>T, p.(Ser619Leu) (S619L)	PH	CNM
	c.1856C>G, p.(Ser619Trp) (S619W)	PH	CNM
	c.1873_1875-delGTC, p.(Val625del) (V625del)	GED	CNM
Echaniz Laguna et al. 2007	c.1102G>C, p.(Glu368Gln) (E368Q)	middle	CNM (+ mild axonal peripheral nerve involvement)
Fabrizi et al. 2007	c.1609G>T, p.(Gly537Cys) (G537C)	PH	CMT2
	c.1709T>A, p.(Leu570His) (L570H)	PH	CMT2
Bitoun et al. 2008	c.1675_1677del, p.(Lys559del) (K559del)	PH	CMT
Claeys et al. 2009; Kontogeorgiou et al. 2023	c.1072G>A, p.(Gly358Arg) (G358R)	middle	CMT (+ neutropaenia and cataract); CMT2, axonal neuropathy
Claeys et al. 2009	c.2564_2569delCCATTA, p.(Thr859_Ile860del)	PRD	CMT (+ neutropaenia and cataract)
Bitoun et al. 2009	c.1678G>A, p.(Glu560Lys) (E560K)	PH	CNM
Jungbluth et al. 2010	c.1862T>C, p.(Leu621Pro) (L621P)	PH	CNM
Melberg et al. 2010	c.1853C>A, p.(Ala618Asp) (A618D)	PH	CNM
Susman et al. 2010; Park et al. 2017; Vandersmissen et al.	c.1565G>A, p.(Arg522His) (R522H)	PH	CNM; CMTDIB

2018; Ganaraja Valakunja et al. 2024			
Bohm et al. 2012	c.1885–1893+8del17	GED	CNM
	c.1880C>G, p.(Pro627Arg) (P627R)	GED	CNM
	c.1567A>G, p.(Arg523Gly) (R523G)	PH	CNM
	c.1564C>T, p.(Arg522Cys) (R522C)	PH	CNM
Kierdaszuck et al. 2013	c.2030G>A, p.(Asp614Asn) (D614N)	PH	CNM
Catteruccia et al. 2013	c.1124T>G, p.(Val375Gly) (V375G)	middle	CNM
	c.1618G>A, p.(Glu540Lys) (E540K)	PH	CNM
	c.1872dupAGCTGG (p.624insA-G)	GED	CNM
Park et al. 2014	c.1948G>A, p.(Glu650Lys) (E650K)	GED	CNM
Abath et al. 2015	c.1115T>G, p.(Phe372Cys) (F372C)	middle	CNM
Casar Borota et al. 2015	c.1940C>G, p.(Pro647Arg) (P647R)	GED	CNM
Chen et al. 2015	c.2153G>A, p.(Arg718Gln) (R718Q)	GED	CNM
Antoniadi et al. 2015	c.700G>C; p.(Val234Leu) (V234L)	G	“Complex phenotype”
	c.788C>T, p.(Pro263Leu) (P263L)	G	CMT
	c.1352G>A; p.(Arg451Gln) (R451Q)	middle	CMT
	c.1739T>C; p.(Met580Thr) (M580T)	PH	CMT
O’ Grady et al. 2016	c.1880C>A, p.(Pro627His) (P627H)	PH	Infantile hypotonia, eventration of diaphragm, gross motor delay, facial weakness, ptosis, ophthalmoplegia, contractures. CK normal. Dystrophic muscle biopsy.
Punetha et al. 2016	c.853C>G; p.(Leu285Val) (L285V)	G	Unknown
Biancalana et al. 2018	c.1831G>A, p.(Glu611Lys) (E611K)	PH	CNM
	c.1090C>T, (p.Arg364Cys) (R364C)	middle	CNM
Westra et al. 2019	c.1666G>A, p.(Glu556Lys) (E556K)	PH	CNM
Topf et al. 2020	c.745C>T, p.(Arg249Cys) (R249C)	G	Unknown
Topf et al. 2020; Ferreira et al. 2024 PMID: 38549004	c.1291dup; p.(V431Gfs*52)	middle	Unknown; CMT
Topf et al. 2020	c.1373G>A; p.(Arg458Gln) (R458Q)	middle	Unknown
Narang et al. 2020	c.292C>T; p.(Arg98Trp) (R98W)	G	Unknown
Ferese et al. 2021	c.890G>A; p.(Arg297His) (R297H)	G	CMT
Natera De Benito et al. 2021	c.869G>A p.(Arg290Gln) (R290Q)	G	Unknown clinical phenotype; Histopathological patterns: cores
Reumers et al. 2021	c.1832G>T, p.(Ser611Ile) (S611I)	PH	CNM
	c.1931_1933del, p.(Gln644del) (Q644del)	GED	CNM
	c.2245G>A, p.(Asp749Asn) (D749N)	PRD	mixed CNM/CMT

	c.596G>A, p.(Arg199Gln) (R199Q)	G	CNM
	c.1058C>G, p.(Thr353Ser) (T353S)	Middle	mixed CNM/CMT
Fujise et al. 2022	c.881C>T, p.(Pro294Leu) (P294L)	G	CNM (isolated progressive proximal lower limbs weakness)
	c.1559T>G, p.(Val520Gly) (V520G)	PH	CNM
	c.1483G>A, p.(Gly495Arg) (G495R)	middle	CNM
	c.1871G>T, p.(Gly624Val) (G624V)	GED	CNM
	c.2171G>A, p.(Arg724His) (R724H)	GED	CNM
Hayes et al. 2022	c.1115T>C, p.(Phe372Ser) (F372S)	middle	CNM
Thomas et al. 2022	c.851A>G; p.(Gln284Arg) (Q284R)	G	Unknown
Kontogeorgiou et al. 2023<br>PMID: 37747677	c.2179C>T, p.(His727Tyr) (H727Y) (VUS)	GED	dHMN
Ganaraja Valakunja et al. 2024<br>PMID: 38968056	c.808G>A, p.(Asp270Asn) (D270N) (VUS)	G	Congenital myopathy
Ganaraja Valakunja et al. 2024	c.1622_1627delACTGGT, p.(Tyr541_Trp542del) (Y541_W542del) (VUS)	PH	Histologically CNM
Ganaraja Valakunja et al. 2024	c.2392G>A, p.(Val798Met) (V798M) (VUS)	PRD	Histologically CNM
Cerdà et al. 2024<br>PMID: 3768954	c.1093A>G, p.(Ile365Val) (I365V) (VUS)	middle	hemorrhagic hereditary telangiectasia plus
LOVD database	c.41A>G, p.(Asn14Ser) (N14S)	G	Unknown
LOVD database	c.269A>G, p.(Lys90Arg) (K90R)	G	Unknown
LOVD database	c.637G>T, p.(Gly213Cys) (G213C)	G	Unknown
LOVD database	c.1052T>C, p.(Val351Ala) (V351A)	middle	Unknown
LOVD database	c.1103A>T, p.(Glu368Val) (E368V)	middle	Unknown
LOVD database	c.1384A>G, p.(Thr462Ala) (T462A)	middle	Unknown
LOVD database	c.1456A>G, p.(Ile486Val) (I486V)	middle	Unknown
LOVD database	c.1493A>C, p.(Asn498Thr) (N498T)	middle	Unknown
LOVD database	c.1609G>A, p.(Gly537Ser) (G537S)	PH	Unknown
LOVD database	c.1619A>G, p.(Glu540Gly) (E540G)	PH	Unknown
LOVD database	c.1893+32dup	GED	Unknown
LOVD database	c.2144A>G, p.(Gln715Arg) (Q715R)	GED	Unknown
LOVD database	c.2276G>A, p.(Ser759Asn) (S759N)	PRD	Unknown
LOVD database	c.1932del, p.(Met645Trpfs*39) (M645Wfs*39)	GED	Unknown
Our patient	c.1726G>A, p.(Glu576Lys) (E576K)	PH	CNM

Supplementary Table 2. List of prediction tools used for the classification of the identified *DNM2* variant.

**Predictor**	**Prediction (Score)**	**Website**	**Reference**
MetaLR	Pathogenic supporting (0.8444)	https://arxiv.org/abs/2206.01408	PMID: 25552646
MetaRNN	Uncertain (0.6144)	http://www.liulab.science/metarnn.html	doi.org/10.1101/2021.04.09.438706
REVEL	Uncertain (0.681)	https://sites.google.com/site/revelgenomics/	PMID: 27666373
CADD	Deleterious (23.3)	https://cadd.gs.washington.edu	PMID: 33618777
DANN	Damaging (0.9902)	https://cbcl.ics.uci.edu/public_data/DANN/	PMID: 25338716
DEOGEN2	Uncertain (0.6217)	https://openebench.bsc.es/tool/deogen2	PMID: 28498993
PaPI	Pathogenic	http://papi.unipv.it	PMID: 25928477
FATHMM	Uncertain (-3.69)	http://fathmm.biocompute.org.uk	PMID: 23033316
M-CAP	Pathogenic moderate (0.8358)	https://great-help.atlassian.net/wiki/spaces/MCAP/overview	PMID: 27776117
MutPred	Uncertain (0.608)	http://mutpred2.mutdb.org	PMID: 33219223
Mutation Assessor	Pathogenic Supporting (2.96)	http://mutationassessor.org/r3/	PMID: 21727090
MutationTaster	Disease Causing	https://www.mutationtaster.org	PMID: 24681721
Polyphen-2	Tolerated (0.138)	http://genetics.bwh.harvard.edu/pph2/	PMID: 20354512
SIFT	Not tolerated	https://sift.bii.a-star.edu.sg	PMID: 11337480
